# Diabetes and pulmonary infection: how hyperglycaemia shapes the immune system

**DOI:** 10.1038/s41392-024-01784-6

**Published:** 2024-03-13

**Authors:** Christian Herder, Michael Roden, Nicolas Venteclef

**Affiliations:** 1 Institute for Clinical Diabetology, German Diabetes Center, Leibniz Center for Diabetes Research at Heinrich Heine University Düsseldorf, 40225 Düsseldorf, Germany; 2 German Center for Diabetes Research (DZD), Partner Düsseldorf, 85764 München-Neuherberg, Germany; 3 Department of Endocrinology and Diabetology, Medical Faculty and University Hospital Düsseldorf, Heinrich Heine University Düsseldorf, 40225 Düsseldorf, Germany; 4 Institut Necker-Enfants Malades (INEM), Université Paris Cité, INSERM UMR-S1151, CNRS UMR-S8253, Paris, France

**Keywords:** Endocrine system and metabolic diseases, Infection

In a recent study published in *Nature*, Nobs and colleagues aimed to identify novel mechanisms that may explain why diabetes is associated with an increased susceptibility to viral respiratory infections. Their analyses revealed a central role of lung dendritic cells (DC) which exhibited several functional defects induced by hyperglycaemia and consequently result in impaired antiviral immune responses.^1^

Both type 1 and type 2 diabetes are tightly linked to several classical complications such as cardiovascular diseases, chronic kidney disease, peripheral and autonomic neuropathy as well as retinopathy, whereas the association with infections, although well-known and clinically relevant, has been less well studied. Investigations in multiple cohorts and populations have demonstrated that people with diabetes have a higher risk of infection-related complications such as hospitalization, and also an increased risk of post-operative infections compared to people without diabetes. This relationship has recently received considerable attention because people with diabetes have been disproportionally affected by the COVID-19 pandemic. The presence of diabetes itself, but also its comorbidities and specific risk phenotypes such as subclinical inflammation, exacerbates the course of SARS-CoV-2 infection and leads to a higher risk of COVID-19-related death than in people without diabetes.^2^ This observation has prompted subsequent mechanistic studies to better understand why diabetes and the severity of viral infections are so intricately linked.

Nobs et al.^1^ present a significant contribution to this field by studying several diabetes mouse models and using different viral pathogens (influenza A, mouse pneumonia virus) causing pulmonary infections. Compared to normoglycaemic control mice, hyperglycaemic mice showed higher viral titres, a dysregulated adaptive immune response and increased mortality indicating an impairment in antiviral immunity. In order to characterize through which mechanisms hyperglycaemia affects the course of infection, cellular, proteomic, transcriptomic and genomic alterations were assessed. Among the multiple changes present both in steady-state (i.e. in the absence of viral infection) and during viral infection, major reductions in several subsets of DC in the lung were observed. Additionally, lung DC showed several functional impairments. Reduced expression of costimulatory molecules (CD40, CD80, CD86) in lung DC, which are required for antigen presentation and T-cell activation, was related to reduced T-cell expansion. Lung DC showed lower lactate production and higher acetyl-CoA levels but unchanged mitochondrial respiration during hyperglycaemia, indicating an impact of hyperglycaemia on lung DC glycolysis and downstream metabolic pathways. The increase in acetyl-CoA may have promoted epigenetic changes favouring a shift from methylation to acetylation with potential implications for global chromatin accessibility which might affect specific inflammatory loci. It is important to note that the aforementioned effects of hyperglycaemia could be abrogated on multiple levels, e.g. by insulin treatment to lower glucose levels or by inhibition of excessive histone acetylation.

Overall, this study highlights the central role of lung DC for the susceptibility to pulmonary viral infections. From the mechanistic perspective, the results suggest a causal link between hyperglycaemia, epigenetic and immunometabolic changes in lung DC, reduction in antiviral T cell activity and enhanced morbidity and mortality. The study clearly demonstrates that high glucose levels are a major determinant of lung DC function and provides a novel concept of how chronic hyperglycaemia can impair host defence against viral pathogens. In this context, analyses of the German Diabetes Study (GDS) showed that even people with recent-onset diabetes present with impaired spiroergometry-assessed lung function, which correlated with less glucometabolic control, highlighting the role of glucose toxicity as an important mechanism of interorgan communication also for pulmonary diseases.^3^ From a therapeutic perspective, these data reiterate the relevance of glucose control in people with diabetes to mitigate the detrimental impact of infections. They also point towards the possibility of targeting acetylation in the acute setting of pulmonary infections but this needs corroboration by subsequent studies.

One strength of the study is the use of different murine diabetes models for type 1 and type 2 diabetes to investigate the impact of hyperglycaemia. However, it is not straightforward to extrapolate findings from these models to human diabetes. The extent and the duration of hyperglycaemia in people with diabetes that would be required for the aforementioned detrimental effects on lung DC in mice are unclear. Moreover, the risk of infections and other comorbidities of diabetes is probably also influenced by other alterations in the immune system. Recent advances to better understand the heterogeneity of diabetes suggested the existence of novel subtypes that differ in immunological characteristics such as biomarkers of inflammation and leucocyte frequencies in blood.^4,5^ Given their differences in glycaemic, non-glycaemic and immunological determinants, it is conceivable that the novel diabetes subtypes also differ in their susceptibility to viral infections. The identification of those at highest risk merits further investigation. Finally, it is most likely that different viruses may act through different mechanismus in their impact of the immune system in the presence of hyperglycaemia, so that the present study could guide future investigations in this field.

**Fig. 1:**
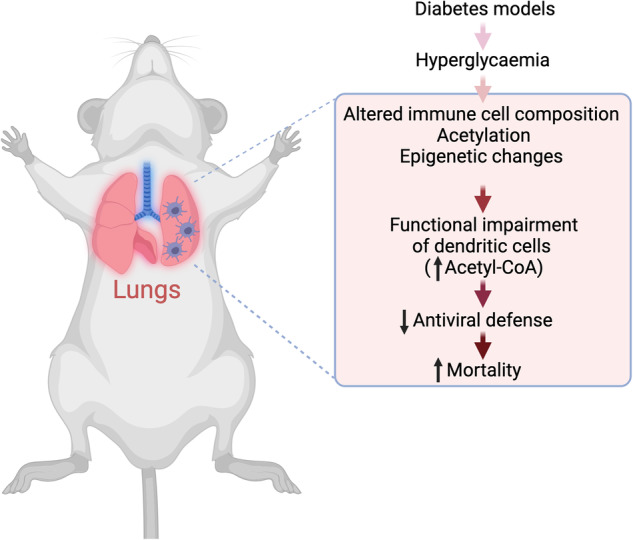
Hyperglycaemia impairs antiviral defence. In a study using several mouse models of diabetes, hyperglycaemia led to complex changes in the lung which impaired antiviral immune responses.^1^ Hyperglycaemia altered the composition of immune cells in the lung, particularly of dendritic cells (DC), and induced epigenetic changes, i.e. increased acetylation. In turn, DC showed functional impairments, including a reduction in the expression of costimulatory molecules that are necessary for antigen presentation to T cells and an increase in acetyl-CoA levels. These changes were related to a less effective antiviral defence and higher virus titres as well as increased morbidity and mortality. The figure was created with BioRender.com

Taken together, Nobs and colleagues provide novel insight into the intricate link between hyperglycaemia, immunity and the risk related to viral infections (Fig. 1). The data suggest that epigenetic alterations could be major determinants of impaired lung DC function and in consequence reduced T-cell mediated antiviral response with crucial impact on morbidity and mortality. While the relevance of adequate glucose-lowering treatment in people with diabetes is the most obvious conclusion, the study also once more highlights the complex interplay between metabolism, the immune system and disease risk.
